# Bioepoxy based hybrid composites from nano-fillers of chicken feather and lignocellulose Ceiba Pentandra

**DOI:** 10.1038/s41598-021-04386-2

**Published:** 2022-01-10

**Authors:** Sanjay Mavinkere Rangappa, Jyotishkumar Parameswaranpillai, Suchart Siengchin, Mohammad Jawaid, Togay Ozbakkaloglu

**Affiliations:** 1 Natural Composites Research Group Lab, Department of Materials and Production Engineering, The Sirindhorn International Thai-German Graduate School of Engineering (TGGS), King Mongkut’s University of Technology North Bangkok, Bangkok, Thailand; 2 Mar Athanasios College For Advanced Studies Tiruvalla (MACFAST), Pathanamthitta, Kerala India; 3 Department of Biocomposite Technology, Institute of Tropical Forestry and Forest Products (INTROP), Universiti Putra Malaysia, 43400 UPM Serdang, Selangor Malaysia; 4 Department of Civil Engineering, Ingram School of Engineering, Texas State University, San Marcos, Texas USA

**Keywords:** Engineering, Materials science

## Abstract

In this work, fillers of waste chicken feather and abundantly available lignocellulose Ceiba Pentandra bark fibers were used as reinforcement with Biopoxy matrix to produce the sustainable composites. The aim of this work was to evaluate the mechanical, thermal, dimensional stability, and morphological performance of waste chicken feather fiber/Ceiba Pentandra bark fiber filler as potential reinforcement in carbon fabric-layered bioepoxy hybrid composites intended for engineering applications. These composites were prepared by a simple, low cost and user-friendly fabrication methods. The mechanical (tensile, flexural, impact, hardness), dimensional stability, thermal stability, and morphological properties of composites were characterized. The Ceiba Pentandra bark fiber filler-reinforced carbon fabric-layered bioepoxy hybrid composites display better mechanical performance compared to chicken feather fiber/Ceiba Pentandra bark fiber reinforced carbon fabrics layered bioepoxy hybrid composites. The Scanning electron micrographs indicated that the composites exhibited good adhesion at the interface of the reinforcement material and matrix system. The thermogravimetric studies revealed that the composites possess multiple degradation steps, however, they are stable up to 300 °C. The thermos-mechanical studies showed good dimensional stability of the composites. Both studied composites display better thermal and mechanical performance compared to neat bioepoxy or non-bioepoxy thermosets and are suitable for semi-structural applications.

## Introduction

The growing environmental and economical awareness has driven efforts for the expansion of new materials for various applications. The fiber (i.e., natural or synthetic fibers) based materials have unique advantages of high strength, high stiffness, lightweight, and non-corrosive properties^1–4^. Unfortunately, the materials based on synthetic fibers have serious environmental impacts, caused by their being non-renewable, non-recyclable, and non-degradable, also, their energy-intensive manufacturing process and the health risks caused by them are disadvantages^5–8^. The environmental awareness and legislation, even due to consumer demands in many countries, have increased the pressure on manufacturers to reduce the ecological footprint of their products at all stages of their life cycle^9,10^.

Currently, chicken feathers are disposed of by various poultry industries. Globally, these poultry processing industries generate huge amounts of feather waste, and this waste is a major issue as it causes severe problems to the environment and living beings^11–13^. Another critical issue in the disposal of this waste is to find suitable locations for the disposal areas and to meet the strict environmental quality requirements enforced by the waste management and disposal laws^14^.

Ceiba Pentandra is a member of the Malvaceae family and grows in various countries like India, Thailand, Indonesia, and the United States of America. Kapok is the common name of the Ceiba Pentandra tree and it is cultivated for seed fiber. The cellulose, hemicellulose, lignin, wax, and ash content of Ceiba Pentandra fibers (CPF) are 60.9, 17.5, 23.5, 0.4, and 1.1 percent, respectively^15^. Recently Venkata Reddy et al. studied the tensile and hardness properties of polyester hybrid composites manufactured by combining kapok and glass fibers. The authors observed significant improvement in the tensile and hardness properties of the hybrid composites compared to single fiber-reinforced composites^16^.

Recent studies have shown that chicken feather fibers (CFF) can be used as a reinforcement material to produce polymer composites^17^. Cheng, et al. reported the thermomechanical properties of CFF/PLA green composites processed using the twin-screw-extruder and an injection molding machine. The authors observed a significant increase in tensile and storage modulus by the incorporation of CFF in the PLA matrix^18^. Uzun, et al. observed good improvement in the impact strength with the incorporation of CFF in vinyl ester and polyester matrix, however, the tensile and flexural properties of vinyl ester and polyester matrix is reduced with the incorporation of CFF. Based on the results the authors recommend the manufacturing of the composites with high impact strength by the waste utilization of CFF^19^. Similarly, Bansal, et al. studied the water absorption behavior of CFF reinforced with epoxy composites. The density of the composites is reduced, the void fraction, water absorption, and thickness swelling are marginally increased with the addition of the CFF^20^. In recent work, Reddy, et al. showed that the keratin from chicken feather can be used as an effective sizing agent on polyester and polyester/cotton blends^21^.

This study aims to develop new advanced materials intended for semi-structural applications to help solve the ecological, economical, and environmental problems. Bioepoxy resin is referred to as an environmentally friendly epoxy resin, derived mostly from plant resources^22^. Ceiba Pentandra trees are abundantly grown in India and have low cost, lightweight, and are eco-friendly. Because of their renewability and availability, Ceiba Pentandra fibers were used as reinforcement for the bioepoxy matrix in this study. The above summarized literature has shown that chicken feather fibers have recently attracted significant attention for different engineering applications. Although several studies have been reported on the structure and properties of chicken feather fibers and other natural fibers reinforced composites. Currently, there are no studies on the physical, mechanical, and thermal properties and morphological structure of composites prepared using chicken feather fiber/Ceiba Pentandra bark as potential reinforcement in bioepoxy composites with carbon layer composites for semi-structural applications.

## Materials

The chicken feathers were collected from local poultry farm. The collected feathers were sterilized using alcohol for 24 h, washed in an organic solvent soluble in water (please name the organic solvent used), and dried in an air oven (Make & Model: FRANCE ETUVES, XU058) at 60 °C for 24 h. The fibers having a length of ~ 10 to 30 mm was separated from the quill. Later, Chicken feather fiber (CFF) were converted into a filler form (~ 100 Microns) by using a universal cutting mill (Make & Model: FRITSCH, PULVERISETTE 19). The density of CFF was 0.35–0.40 gm/cc.

The matured barks of Ceiba Pentandra were collected from private property, Bangalore, India (No permission required). Ceiba Pentandra fibers (CPFs) were immersed in water for two weeks to allow retting. Following this, the CPF were extracted using ultrafine long metal teeth. The extracted fibers were then treated with 5% (w/v) sodium hydroxide solution at room temperature for 30 min. Lastly, the treated CPFs were neutralized (please name the neutralizing agent used), cleaned, and dried^23^. Later, chemically treated fibers were converted into filler form (≈500 Microns) by using the Universal Cutting Mill. The density of CPF was 0.55–0.60 gm/cc.

**Figure 1:**
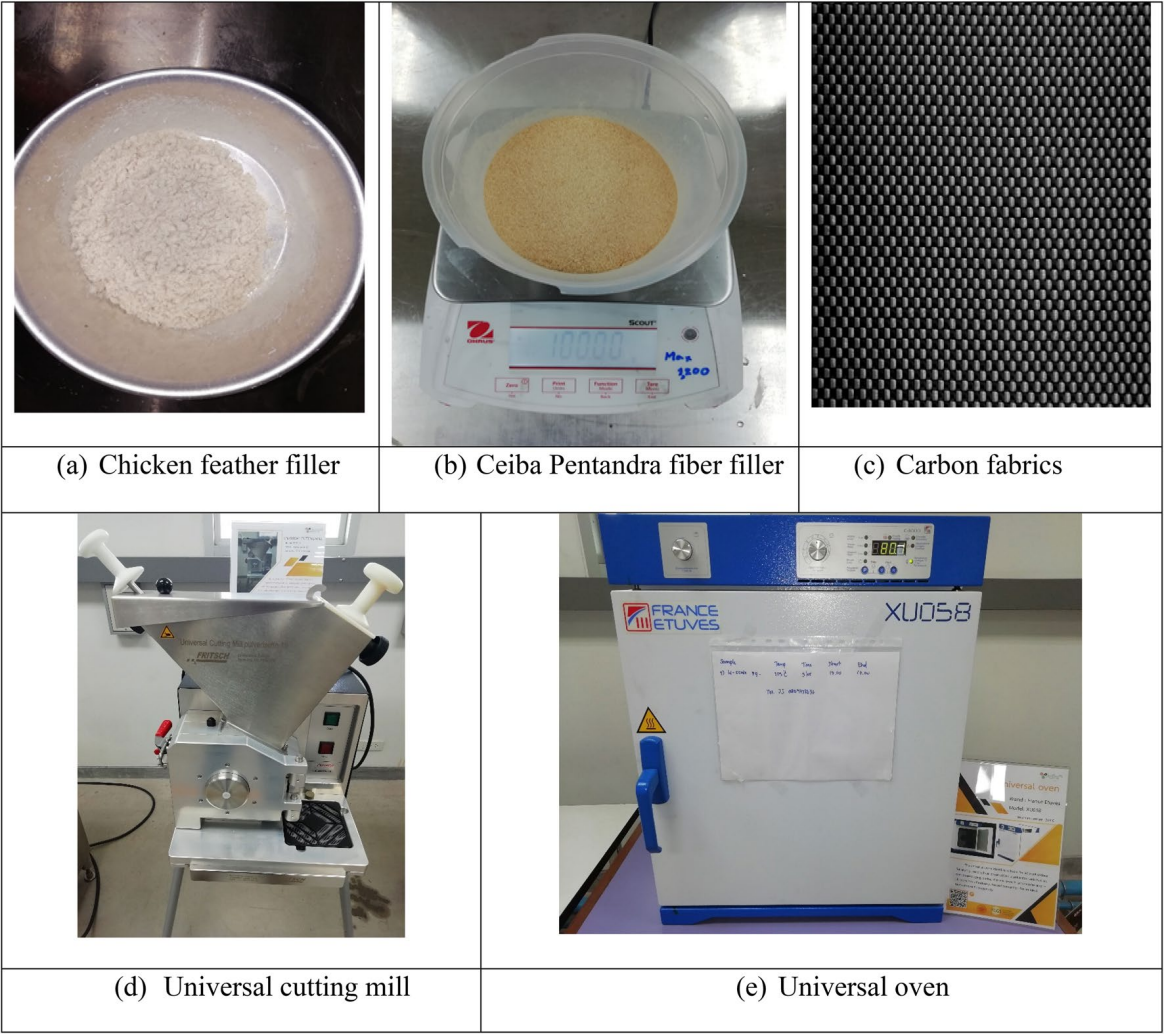
Materials used in this study and the equipment used for processing fibers.

Carbon fabrics in plain-woven form of 360 gsm were used. The bioepoxy resin (GreenPoxy 56′) and curing agent (SD Surf Clear) produced by Sicomi, France, supplied by Cobra International Co Ltd, Chonburi, Thailand, were used. The tensile strength, tensile modulus, and flexural strength of the bioepoxy resin were 53 MPa, 981 MPa, and 58 MPa, respectively. For the preparation of the matrix system, the bioepoxy resin and curing agent were mixed at a ratio of 9:3.333. The pictorial images of the materials and equipment are shown in Fig. 1.

## Experimental study

### Manufacturing of composites and preparation of specimens

**Figure 2:**
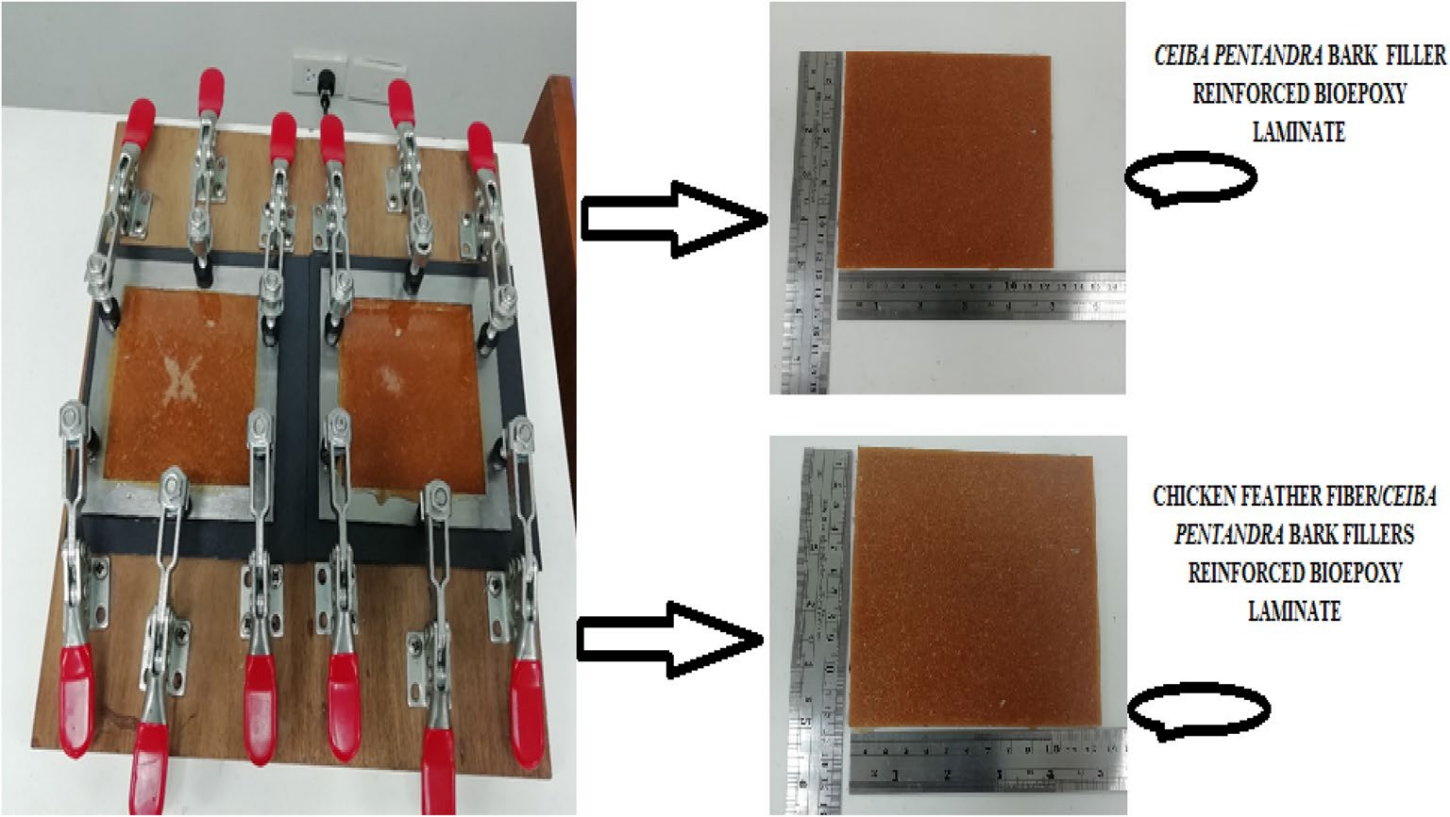
Preparation of laminates by casting method.

Initially, CFF/CPF filler-reinforced bioepoxy laminates were prepared using the solution-casting technique (Fig. 2) as per the weight fractions given in Table 1. The both fiber fillers were mixed with bioepoxy under constant stirring on magnetic stirrer-hotplate at 60 °C for 30 min. The stoichiometric amount of curing agent was then added to the mixture at room temperature and mixed well for 2–3 min. After proper mixing, the laminates were obtained by pouring the solution onto the casting mold as a casting surface and allowing this solution to cure at room temperature overnight. The dimensions of the prepared laminates were 200 × 200 mm at 2.8 mm thickness.

**Table 1:** Composition of composites.

Composites	Weight (g)				Weight fraction (%)				Volume fraction (%)
	w_f_			w_m_	W_f_			W_m_	
	w_CFF_	w_CPF_	w_C_		W_CFF_	W_CPF_	W_C_		
CPCEFC	_-_	100	25	150 ± 5	-	36.36	9.09	54.54	60
CPCFCEFC	50	50	25	150 ± 5	18.18	18.18	9.09	54.54	65

w_f_ is the weight of fiber {weight of Chicken feather fiber (w_CFF_), weight of Cieba Patendra fiber (w_CPF_), and w_m_ is the weight of matrix.

W_f_ is the fiber weight fraction {W_CFF_ is the weight fraction of Chicken feather fiber and W_CPF_ is the weight fraction of Cieba Patendra fiber}, and W_m_ is the matrix weight fraction.

*CPCEFC* Ceiba pentandra bark fiber filler-reinforced carbon fabric-layered bioepoxy composites, *CPCFCEFC* Ceiba pentandra bark fiber/chicken feather fiber filler-reinforced carbon fabric-layered bioepoxy composites. *CFF* Chicken feather fiber, *CPF* Ceiba Pentandra bark fiber, *C* Carbon fabric, *m* Matrix.

**Figure 3:**
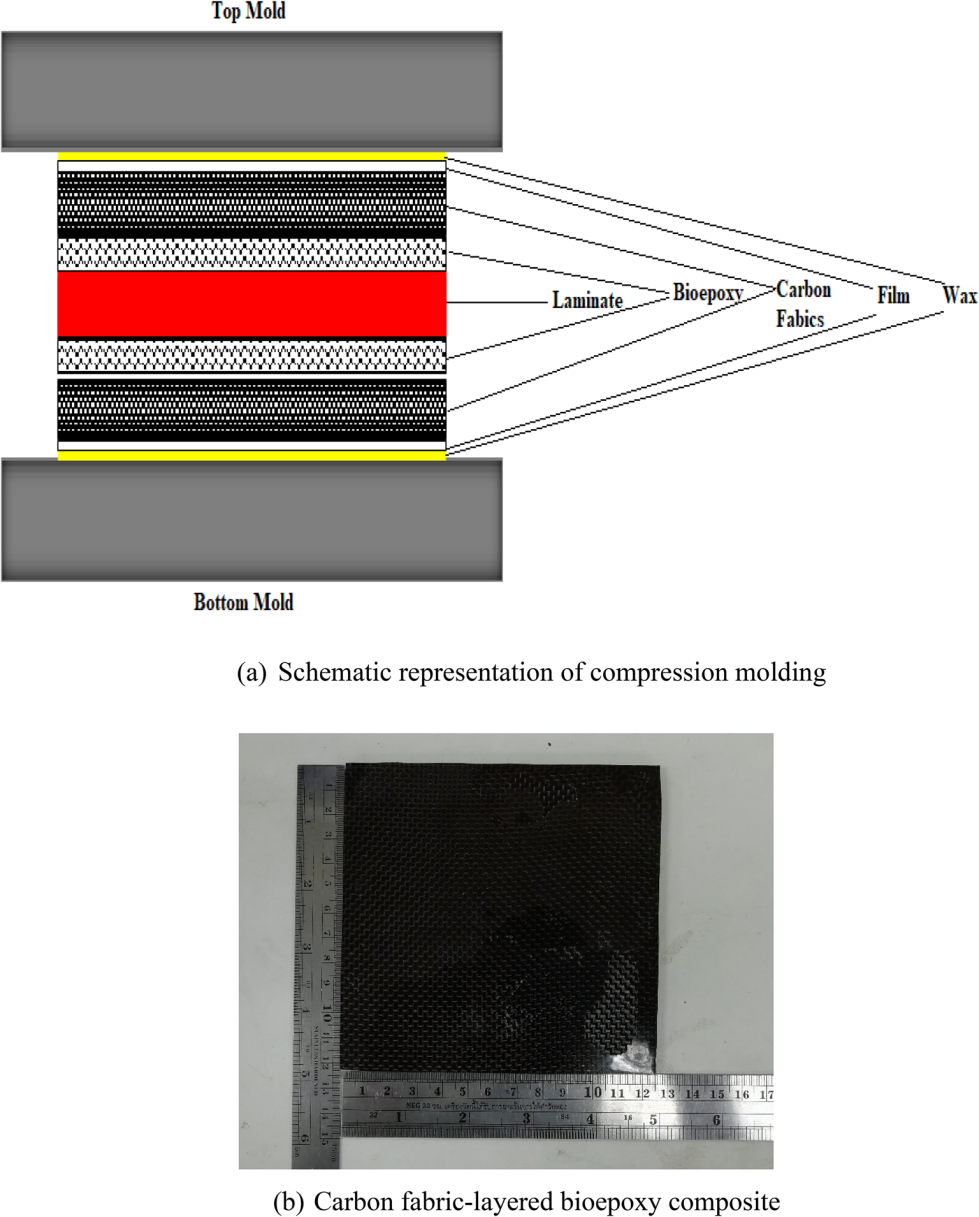
Mold used for layering carbon fabrics as skin layers in laminates.

After casting, the composites were prepared by compression molding (Fig. 3). In this process, the laminate prepared through the casting technique was placed between the carbon fabrics. The bottom mold was covered by thin film sheet and silicone spray was applied over it to avoid adhesion between composite laminate and a mold surface. Then the bioepoxy matrix was distributed evenly after placing carbon fabrics as skin layers (top and bottom layers) on the laminates by using hand lay-up blades. Then, the thin film sheet was placed on the prepared composite laminate to avoid adhesion with the top mold. After this, the laminate was covered with top mold and cured at room temperature for 24 h. Finally, the pre-cured composites (final dimensions: 200 × 200 × 3 mm^3^) were exposed to post-curing in an oven at 80 °C for 24 h. The prepared composites are taken out from the oven and then specimens of suitable dimensions were prepared from composites using a diamond-tipped saw cutter for testing in accordance with ASTM standards. The emery sheets were used to remove rough edges of test specimens. Five nominally identical test specimens were prepared for each different test.

### Testing and characterization

‘ASTM D2734-94’^24^ standard was used to determine voids in composites. The tensile tests were carried out on a Comtech tensile testing machine M1 type universal testing machine (UTM), with a load cell of 1 kN. The dimension, gauge length, and crosshead speed for the tensile test was as per ASTM D3039^25^ standard. The flexural test was conducted using the flexural test fixture of the same UTM at a loading rate of 2.5 mm/min according to ASTM D790-07^26^. The impact resistance of the composites was measured using a pendulum impact tester (Model: Zwick/Roell, 727,676) as per ASTM D256-06^27^ standard. The micro-hardness of the composites was measured on a Shore hardness tester (Model: Rex durometer, OS-1) as per ASTM E384^28^. Thermogravimetric measurements were made in the temperature range of 50 to 700 °C at the heating rate of 10 °C min^−1^ using a TGA 2 (Model: Mettler Toledo TGA/DSC 3+ HT/1600, Switzerland). The fractured surfaces of the test specimens of composites were taken using FEI Quanta 450 scanning electron microscope at an accelerating voltage of 10 kV. The thermomechanical properties of composites were measured using a TA Instruments Q 400 thermomechanical analyzer. Rectangular samples of 20 × 10 × 3 mm^3^ were used for testing. The measurements were made in the temperature range of -50 to 150 °C.

## Results and discussion

### Void content

**Figure 4:**
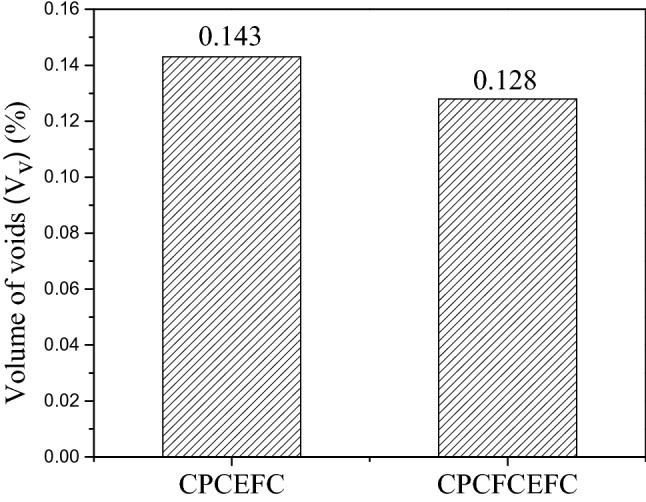
Volume of voids in CPCEFC and CPCFCEFC.

It is known that the mechanical performance of the composites is influenced by their void content, and the presence of voids leads to a reduction in the mechanical and physical properties^29,30^. A good fiber matrix adhesion and fiber wetting will result in a low void content and better mechanical performance^31,32^. Figure 4 shows the volume of voids in the CPCEFC and CPCFCEFC based composites. The calculated volume of voids in the CPCEFC and CPCFCEFC were 0.143 and 0.128, respectively. The low value of the void fraction of the composites suggests uniform filler dispersion, good wetting and interfacial interaction of the bioepoxy resin with the filler and fabrics^33,34^. This indicates that the two-step process (casting technique and the compression molding technique) used in the preparation of the composite samples is highly effective for the manufacturing of advanced composites^35^. In this method, the resin spread and impregnated over fibers and fabrics by removing the air or voids present in the sample, resulting in composites with a lower void fraction.

### Mechanical properties

#### Tensile properties

**Figure 5:**
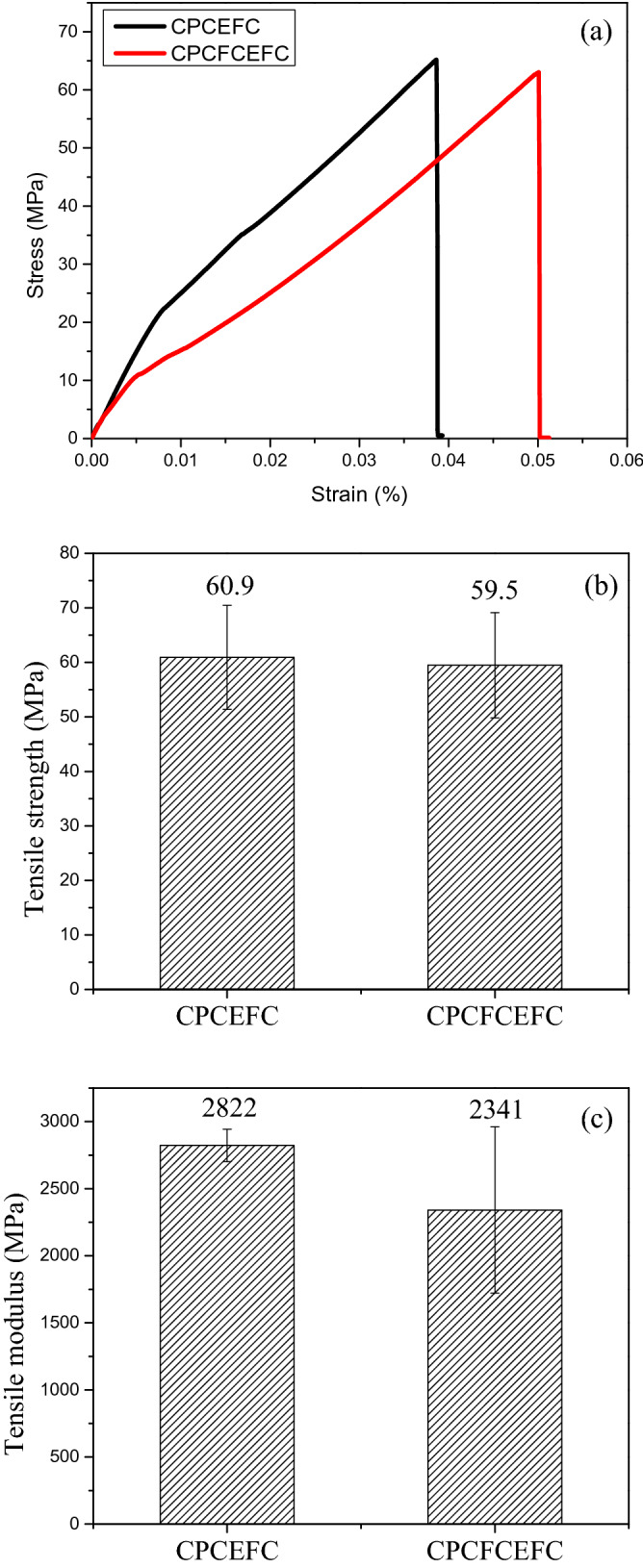
(**a**) Stress–strain plot (**b**) tensile strength, (**c**) tensile modulus of the CPCEFC and CPCFCEFC.

Figure 5a shows the representative tensile stress–strain curve of CPCEFC and CPCFCEFC samples. The tensile strength and tensile modulus of CPCEFC and CPCFCEFC composites are presented in Fig. 5b, c. As be seen, CPCEFC exhibited a slightly higher tensile strength than CPCFCEFC composites. As expected, the tensile strength of the composites is higher than the values reported for neat non-biodegradable and biodegradable epoxy resins (i.e. 60.9 and 59.5 MPa)^36–38^. The remarkable tensile strength of the polymer composites is due to the good interfacial adhesion between the fiber and the polymer matrix, which leads to a lower void content^39,40^. The tensile modulus of CPCEFC containing only Ceiba patendra fiber filler was obtained as 2822 MPa and that of the CPCFCEFC was recorded as 2341 MPa. The incorporation of Ceiba patendra and chicken feather fibers increased the tensile modulus of the composite by approximately 143%, whereas the incorporation of only Ceiba patendra fibers increased the tensile modulus by approximately 193%.

These results point to the possibility of the utilization of waste CP and CF fibers for the development of hybrid composites, which can be suitable for applications that do not require high load capacities. Thus, the incorporation of CP or CP/CF fibers into the composites can be an attractive alternative to the use of more expensive biodegradable epoxy resin and carbon fabrics in certain applications. It is important to note that the role of carbon fabrics on the significant increase in the tensile properties cannot be neglected^41–43^.

**Figure 6:**
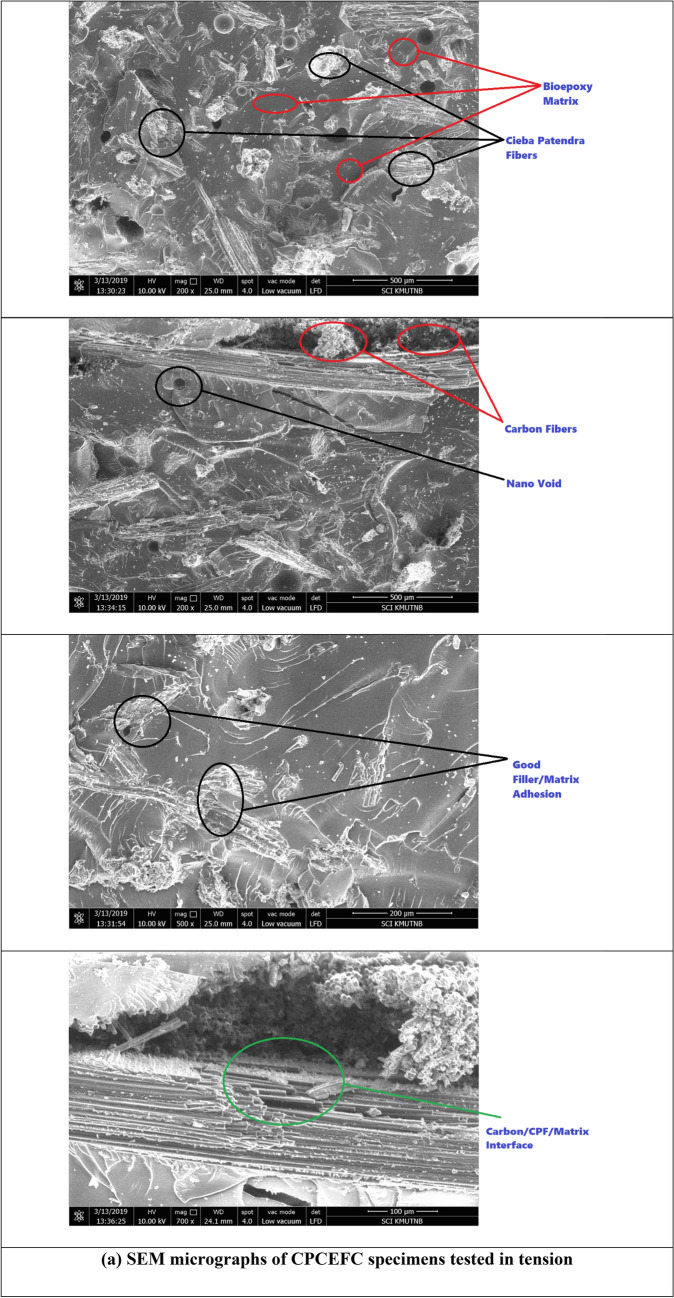

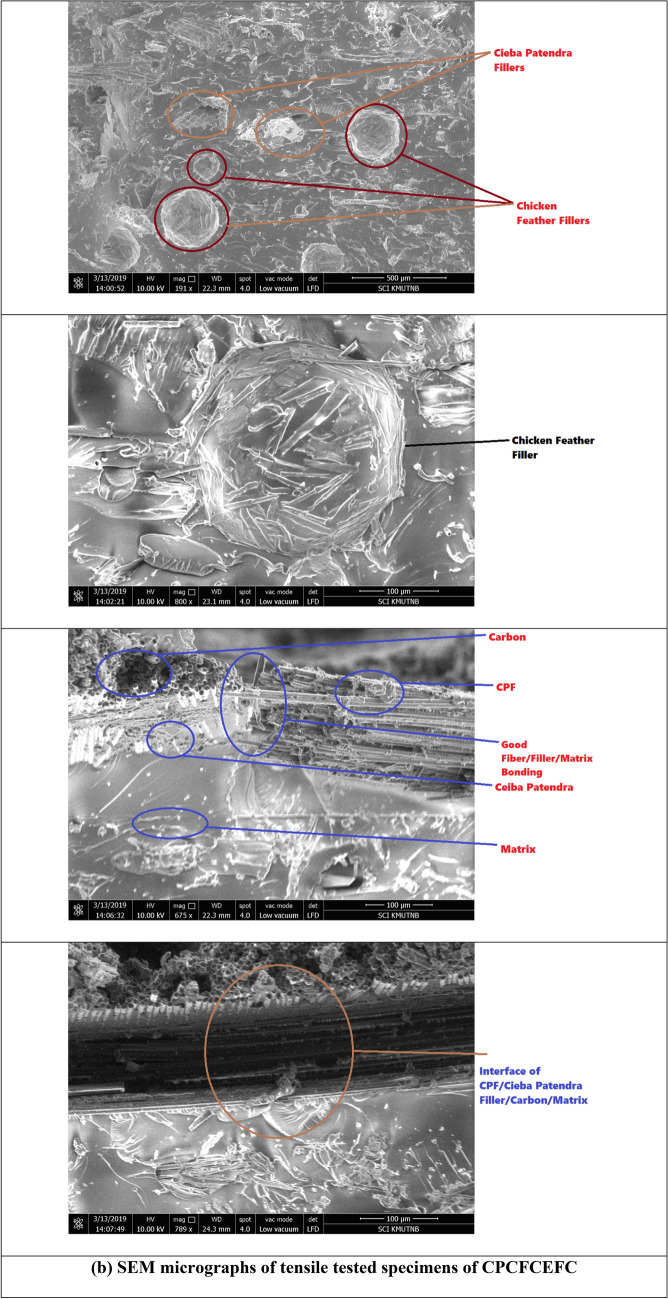
SEM micrographs of tensile tested CPCEFC and CPCFCEFC specimens.

The morphology of tensile fracture surfaces of CPCEFC and CPCFCEFC composites was studied by SEM, and the micrographs at different magnification are shown in Fig. 6. From the micrographs of CPCEFC (Fig. 6a), the CP fibers, carbon fabrics, fiber breakage, fiber matrix debonding, and fiber/matrix interface, are clearly visible. The CP fibers are uniformly distributed within the matrix. The strong fiber matrix interface is observed in the SEM micrographs with few fibers pulling out, which explains the strong fiber matrix interaction. In the case of CPCFCEFC composites (Fig. 6b), big domains of CP and CF fibers are distributed throughout the matrix. Multiple carbon fiber and CF fiber pull-out are also observed in the SEM images. The fiber pull-out suggests the debonding of the fabric from the epoxy matrix at high loads.

#### Flexural properties

**Figure 7:**
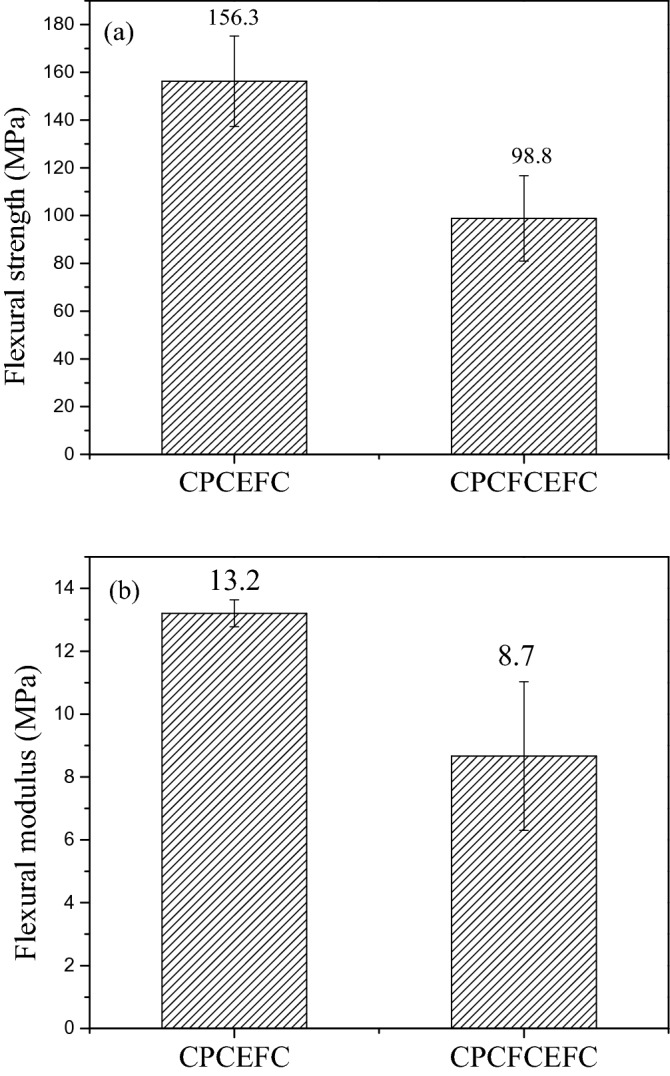
(**a**) Flexural strength and (**b**) flexural modulus of the CPCEFC and CPCFCEFC composites.

**Figure 8:**
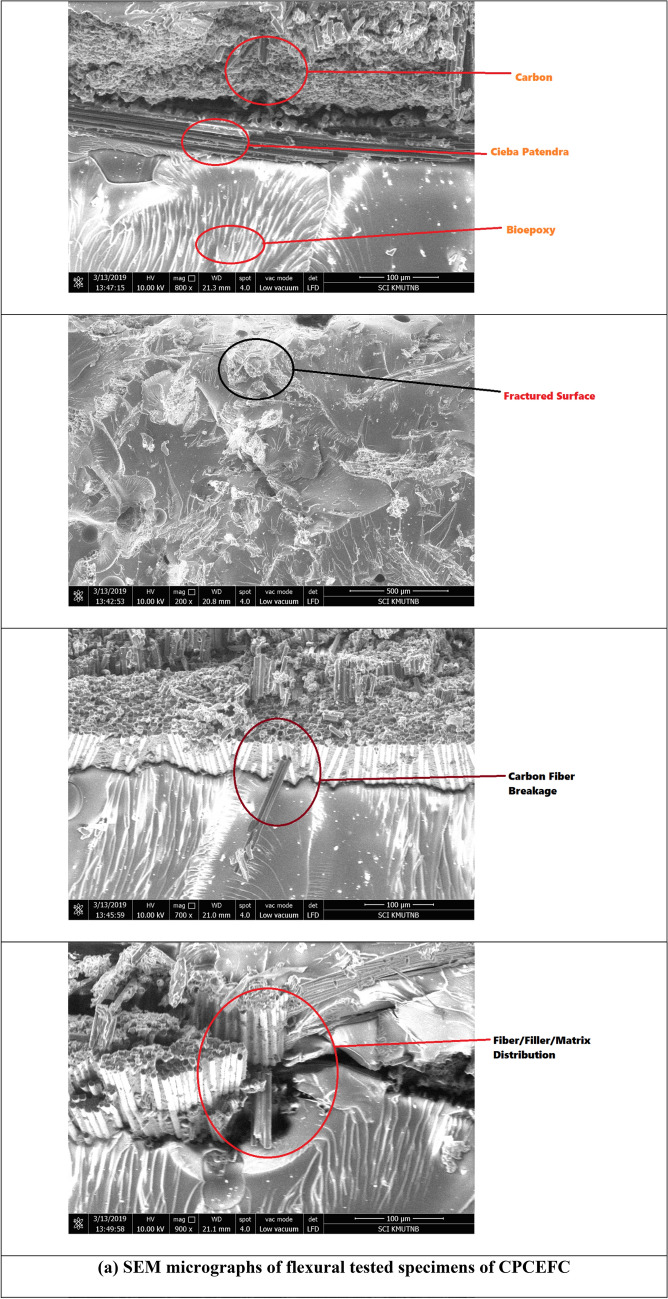

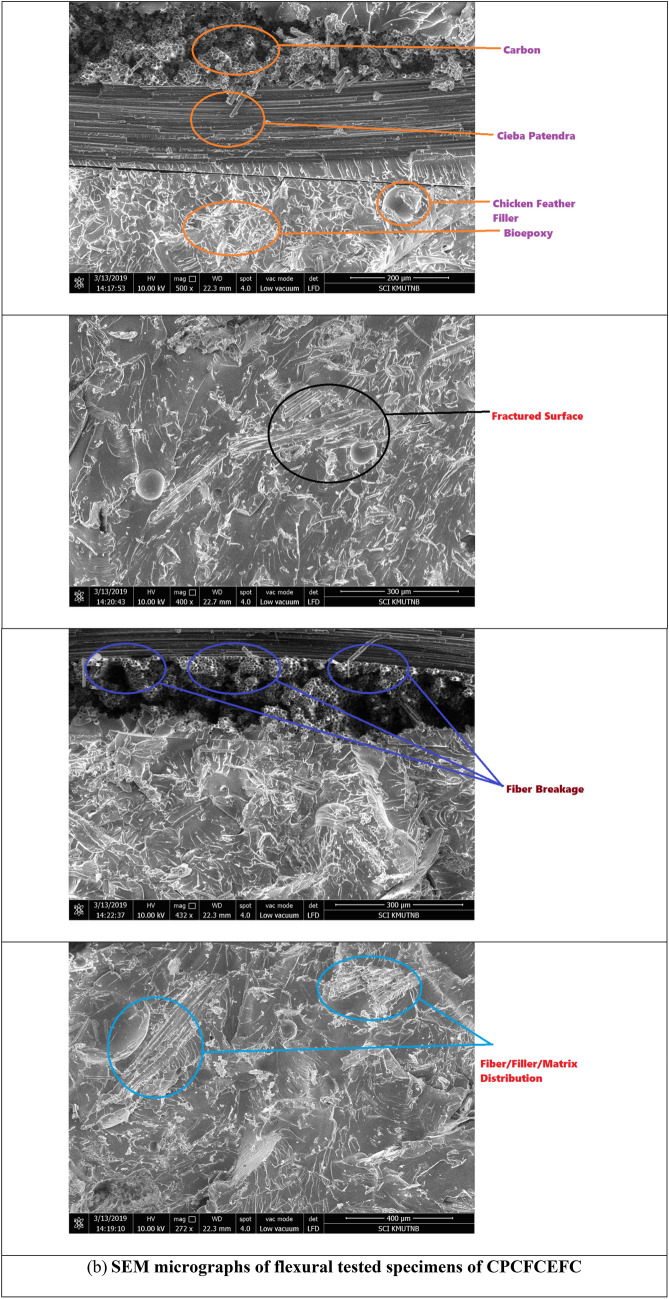
SEM micrographs of flexural tested CPCEFC and CPCFCEFC specimens.

The flexural strength and modulus values of CPCEFC and CPCFCEFC composites are shown in Fig. 7a, b, respectively. The average flexural strength and modulus of CPCEFC composites were 156 MPa and 13.2 GPa, respectively. These values are greater than the values reported for bioepoxy resin and synthetic epoxy resin^44,45^. This could be due to the strong adhesion between the CPF and carbon fabrics and the matrix system. On the other hand, the flexural strength and modulus of CPCFCEFC were 98.8 MPa and ca. 8.67 GPa, respectively. The flexural properties of the composites depend on the matrix deformation before failure. The lower value of flexural strength and modulus values of CPCFCEFC composites could be due to the higher deformation caused at higher loading. It is important to note that the high flexural strength and modulus of the composites was due to the reinforcement imparted by the carbon fibers. The SEM micrographs of CPCEFC and CPCFCEFC specimens after the flexural test are shown in Fig. 8. In both CPCEFC and CPCFCEFC composites, the SEM images show a uniform distribution of fiber and filler in the epoxy matrix system with fiber matrix debonding, and low fiber pull-out. It was observed that no void was found in either composite, and hence in Fig. 8 fracture surface micrographs without voids are presented.

#### Impact resistance

**Figure 9:**
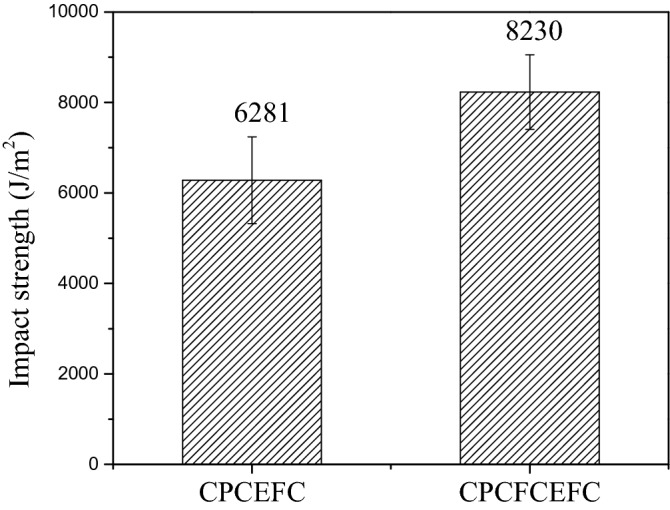
Impact strength of CPCEFC and CPCFCEFC.

**Figure 10:**
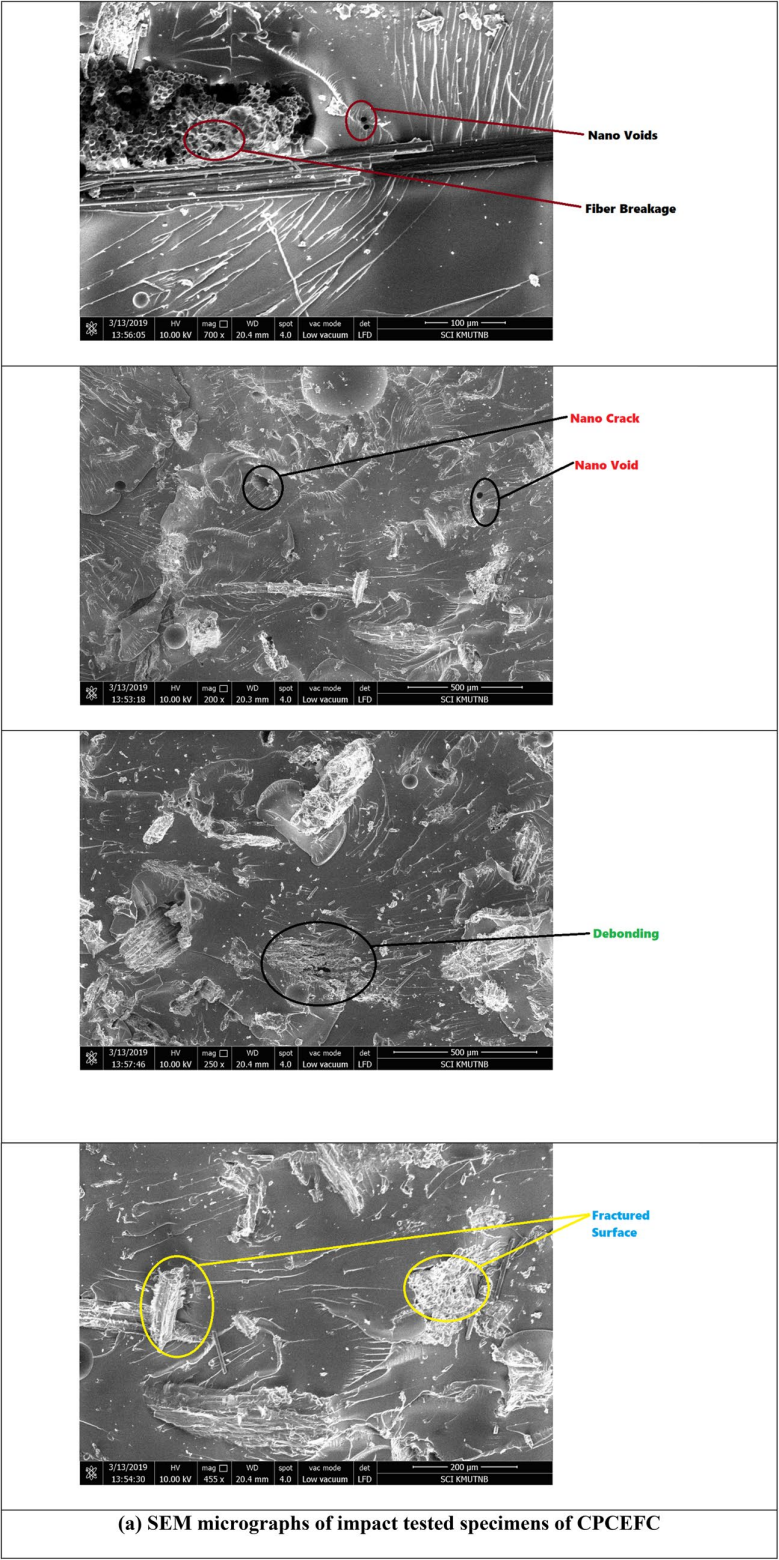

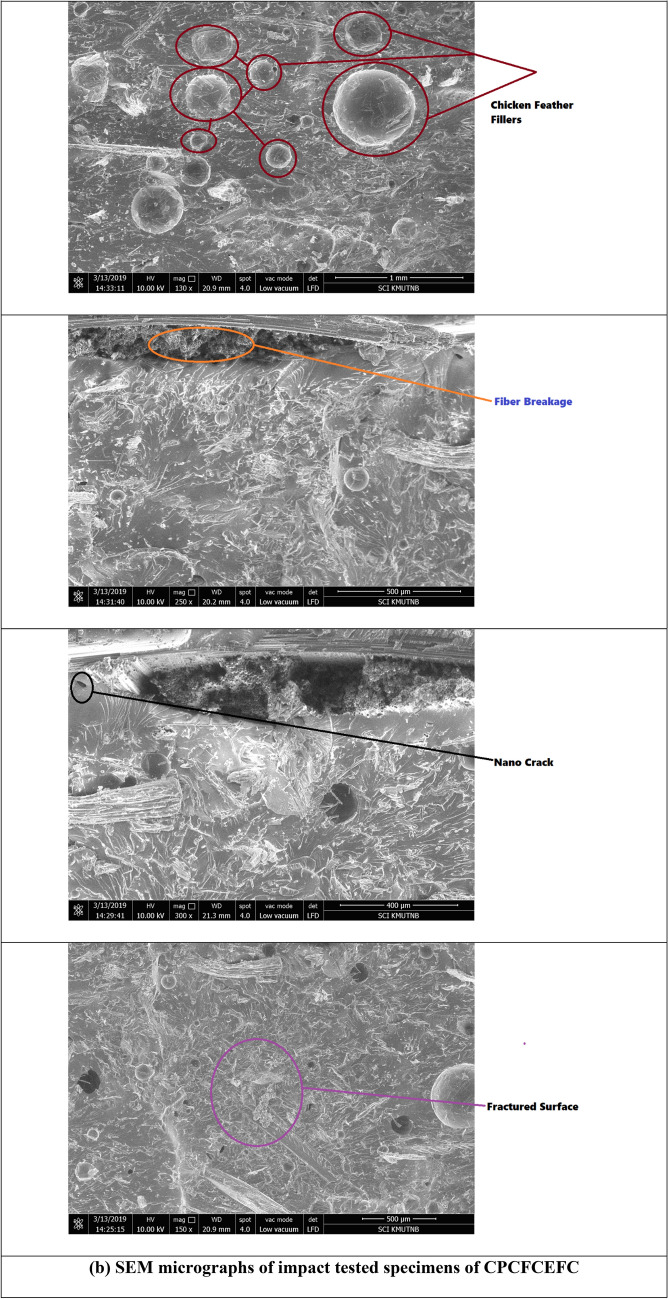
SEM micrographs of impact tested CPCEFC and CPCFCEFC specimens.

Figure 9 presents the impact strengths of the CPCEFC and CPCFCEFC composites. The impact strength of CPCFCEFC was 8229 J/m^2^, whereas that of CPCEFC was 6280 J/m^2^. In addition to the interfacial interaction between the filler and polymer, the impact strength depends on the deformation before failure. The lower impact strength of CPCEFC is as expected since the materials with high strength and modulus undergo a failure at low deformations. The impact strengths obtained for the hybrid composites in this study are greater than the impact strength of the reported values of synthetic non-biodegradable epoxy resin and natural biodegradable epoxy resins^46,47^. The increase in the impact strength is due to the better fiber-matrix interaction. These results indicate that the incorporation of CF fibers is a suitable choice for the improvement of the impact strength. This is because of the flexible nature of the CF fibers, and with their addition the bioepoxy matrix system tends to absorb more impact energy. Figure 10 presented the SEM micrographs for the CPCEFC and CPCFCEFC specimens after the impact test. Fiber failure, voids, fiber pullout, microcracks, and river-like patterns are visible in the SEM micrographs. The composites show low fiber pull out and low void content, indicating the excellent wetting of the fibers with good interfacial adhesion, resulting in the improved interfacial interaction between the fiber and polymers. These are consistent with the understanding that the interfacial adhesion between fiber/filler/matrix determines the impact strength^48,49^.

#### Hardness

**Figure 11:**
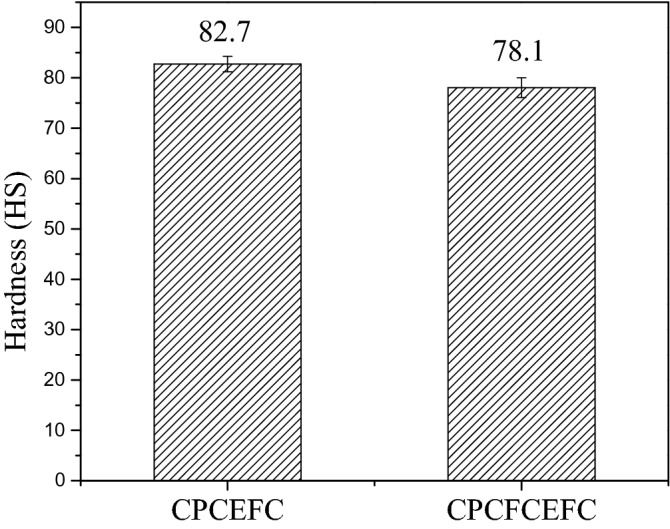
Hardness values of the CPCEFC and CPCFCEFC.

Micro-hardness test results of CPCEFC and CPCFCEFC specimens are shown in Fig. 11. The hardness of CPCEFC was higher than that of CPCFCEFC (i.e. 82.7 HS vs 78.1 HS). These results indicate that the addition of CF fibers reduced the hardness of the composites, and this is in line with the results obtained from tension and flexure tests. The high hardness values obtained in the composites tested in this study is due to the high modulus of carbon fabrics and CP fibers^50–52^.

#### Thermal properties

**Figure 12:**
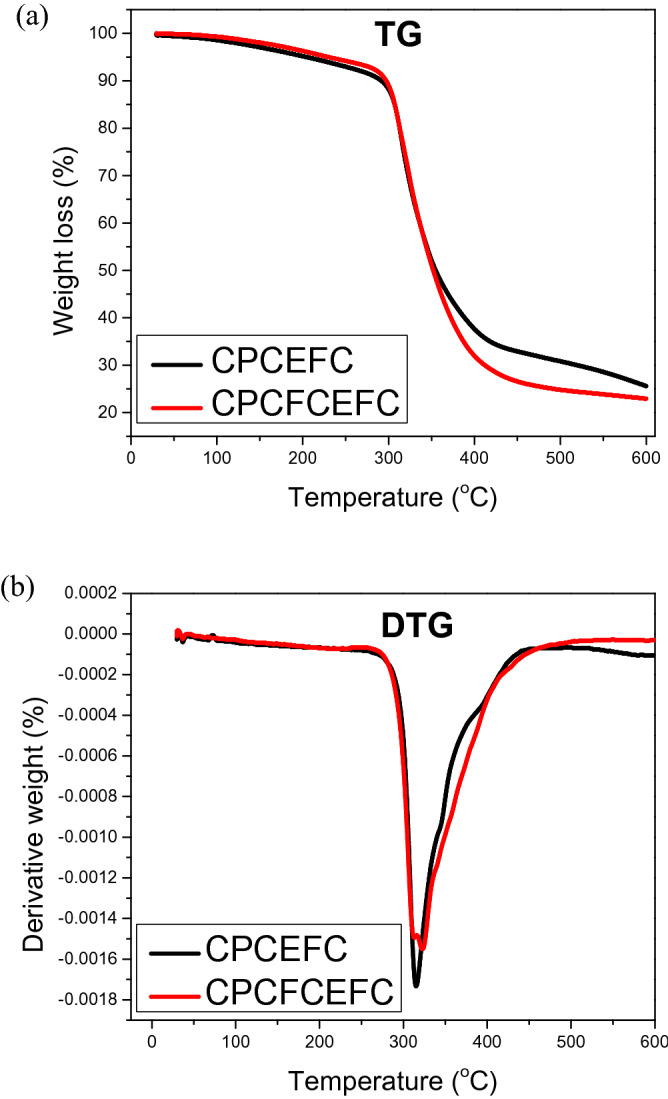
(**a**) TG and (**b**) DTG curve of the CPCEFC and CPCFCEFC.

The thermogravimetric (TG) curve and the derivative of thermogravimetric (DTG) curve are shown in Fig. 12. The degradation of various ingredients of CPCEFC and CPCFCEFC takes place in three phases. The first phase of the degradation occurred at approximately 100 °C due to the evaporation of moisture from the composites^53^. The main degradation at 300 °C was due to the degradation of the epoxy matrix^54^. The degradation above 400 °C was caused by the degradation of carbon fabrics. Both types of composites showed a nearly identical behavior. However, there was a lower amount of char residue in CPCFCEFC compared to CPCEFC due to the presence of CF fibers. Also, the minor degradation observed at 350 °C in CPCEFC composites is attributed to CP fibers^15^. The CF fibers undergo decomposition from 350 °C onwards; however, this is not visible in the TG and DTG curves^18^. The TGA results indicate that these composites are suitable candidates for applications with working temperatures up to 310 °C.

#### Thermomechanical analysis (TMA)

**Figure 13:**
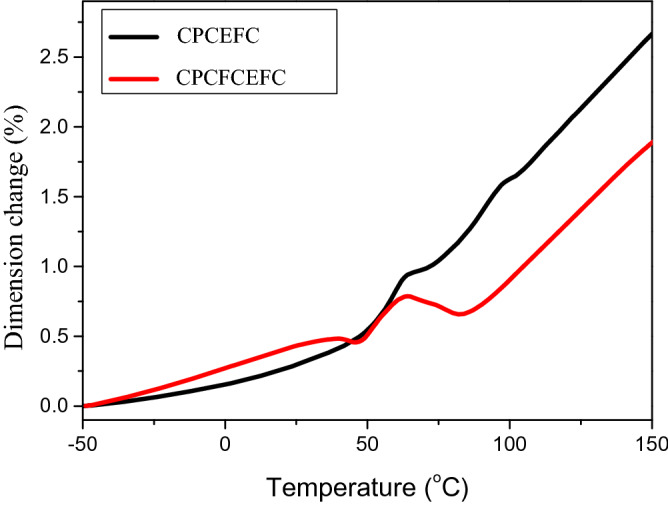
TMA profile of the CPCEFC and CPCFCEFC.

The change in dimension of CPCEFC and CPCFCEFC composites with respect to temperature are shown in Fig. 13. The change in dimension is due to molecular vibrations of the polymer chains, which increases with increasing temperature. TMA profiles of both composites show that the change in dimension slowly increases with increasing temperature up to 50 °C (T_g_ of the epoxy matrix), which is followed by a rapid expansion of the composites at higher temperatures (typical expansion behavior of epoxy thermosets in the rubbery state)^55,56^. Consequently, the transition from the glassy state to the rubbery state is clearly visible from the TMA profile. In the glassy state, the dimensional stability of CPCEFC composites is marginally higher than that of CPCFCEFC composites. However, in the rubbery state CPCEFC composites exhibit greater expansion. That is to say CPCEFC composites are dimensionally more stable in the glassy state, whereas CPCFCEFC composites are more stable in the rubbery state. The dimensional stability of CPCEFC composites is due to its higher crosslink density. In the case of CPCFCEFC, the dilution effect induced by the CF fibers may marginally lower the epoxy amine reaction, and hence the dimensional stability decreases. On the other hand, in the rubbery state, the CF fibers restrict the mobility of epoxy polymer chains, and hence the dimensional stability increases.

## Summary and conclusions

This study has presented a new way to utilize waste Chicken feather fibers and Ceiba Pentandra bark as reinforcement materials in polymer composites. This study has also introduced a novel approach to improve the properties of composites using carbon fabrics as skin layers with plant and animal fibers through the use of compression molding technique. The study has also examined a new idea of introducing bioepoxy resin for manufacturing composites. Hybrid bioepoxy composites of chicken feather fiber/Ceiba Pentandra bark reinforced bioepoxy composites with carbon layers (CPCEFC and CPCFCEFC) were prepared. In mechanical tests, the prepared composites have exhibited good tensile strength, tensile modulus, flexural strength, flexural modulus, and impact strength. The tensile and flexural properties are higher for CPCEFC composites, while the highest impact strength is observed for CPCFCEFC composites. The SEM micrographs confirmed that, the composites have good interfacial interaction between the fiber and matrix with low void fraction and low fiber pull-out. The composites also showed good dimensional stability, especially at lower temperatures. The composites are thermally stable up to 300 °C, hence can be used for high temperature applications. The relatively high mechanical and thermal properties of the prepared composites make the composite material useful for semi-structural applications.
